# Serum levels of protein carbonyl, a marker of oxidative stress, are associated with overhydration, sarcopenia and mortality in hemodialysis patients

**DOI:** 10.1186/s12882-020-01937-z

**Published:** 2020-07-16

**Authors:** Young Rim Song, Jwa-Kyung Kim, Hyung-Seok Lee, Sung Gyun Kim, Eun-Kyoung Choi

**Affiliations:** 1 Division of Nephrology, Hallym University Sacred Heart Hospital, 22, Gwanpyeong-ro 170 beon-gil, Dongan-gu, Anyang, 431-070 Republic of Korea; 2 Hallym University Kidney Research Institute, Anyang, Republic of Korea; 3 Department of Biomedical Gerontology, Graduate School of Hallym University, Chuncheon, Republic of Korea; 4 Ilsong Institute of Life Science, Hallym University, Anyang, Republic of Korea

**Keywords:** Oxidative stress, Hemodialysis, Mortality, Protein carbonyl, Overhydration, Sarcopenia

## Abstract

**Background:**

Increased oxidative stress in end-stage renal disease is regarded as one of the important mechanisms in the atherosclerosis and muscle wasting. However, studies examining the clinical significance of oxidative stress by direct measurement of these markers and its association with volume status and sarcopenia are limited.

**Methods:**

A follow-up cross-sectional study was performed in stable hemodialysis (HD) patients and serum protein carbonyl levels were measured as a biomarker of oxidative stress. Additionally, multi-frequency body composition analysis, handgrip strength (HGS) and nutritional assessments were performed at baseline.

**Results:**

Eighty-eight patients undergoing HD were included and 30 (34.1%) patients died during a mean follow-up of 5.2 years. The mean patient age was 60.6 ± 13.5 years, and the mean HD duration was 50.8 ± 41.3 months. In total, 16 patients (18.2%) were overhydrated, 49 (55.7%) had low HGS and 36 (40.9%) had low muscle mass. Serum protein carbonyl levels were associated with serum levels of albumin, prealbumin and transferrin, hydration status and low HGS. Overhydration (odds ratio [OR] 7.01, 95% CI 1.77–27.79, *p* = 0.006), prealbumin (OR 0.91, 95% CI 0.83–0.99, *p* = 0.030), subjective global assessment (OR 3.52, 95% CI 1.08–11.46, *p* = 0.037) and sarcopenia (OR 3.41, 95% CI 1.02–11.32, *p* = 0.046) were significantly related to increased serum protein carbonyl levels. Multivariate analysis showed that the serum levels of protein carbonyl (Hazard ratio [HR] 2.37, 95% CI 1.02–5.55, *p* = 0.036), albumin (HR 0.17, 95% CI 0.06–0.46, *p* = 0.003), prealbumin (HR 0.86, 95% CI 0.80–0.92, *p* = 0.001), overhydration (HR 2.31, 95% CI 1.26–8.71, *p* = 0.015) and sarcopenia (HR 2.72, 95% CI 1.11–6.63, *p* = 0.028) were independent determinants of all-cause mortality.

**Conclusions:**

Serum protein carbonyl was significantly associated with overhydration, nutritional status and sarcopenia, and could be a new predictor of mortality in patients undergoing HD.

## Background

Patients with end-stage renal disease (ESRD) have higher mortality and morbidity than the age-matched general population, with cardiovascular disease and infection being the major causes of mortality [1, 2]. This increased mortality cannot be fully explained by a higher prevalence of traditional risk factors, such as diabetes, hypertension, hypercholesterolemia, and physical inactivity. Additionally, uremia-specific factors such as inflammation, protein-energy wasting, and oxidative stress might contribute to the excessive mortality risk in these patients [3–6]. Uremia and hemodialysis (HD) contribute to an increase in free radical production and a decrease in antioxidant levels in patients undergoing HD [7–9]. Studies have demonstrated that biomarkers of oxidative stress are significantly increased and associated with surrogate markers of atherosclerosis such as endothelial dysfunction, vascular or coronary calcification, amyloidosis, intima-media thickness, left ventricular hypertrophy, and anemia in patients with ESRD [10–13]. Oxidative stress is increased in the early stages of chronic kidney disease (CKD) [14–17] and continues to gradually increase in later stages of CKD, becoming more severe with HD procedures [18]. Besides the HD process itself, several dialysis-related factors play an important role on oxidative stress in HD. Duration of dialysis, iron administration, anemia, the presence of central venous catheter, the type and malfunctioning of vascular access, type of dialyzer membranes, HD modality, and anticoagulation are involved in the pathogenesis of oxidative stress in HD patients [9, 19].

In this study, we examined protein carbonyl as a biomarker of oxidative stress, which has some advantages because of the relatively early formation and stability of carbonylated proteins [20, 21]. Previous studies have demonstrated the close relationships between oxidative stress, inflammation, hypoalbuminemia and malnutrition [22, 23], but the role of oxidative stress as a predictor of long-term outcomes and its relationships with volume status and muscle mass and strength in HD patients remain controversial and uncertain [8, 24]. We hypothesized that overhydration, malnutrition, and low muscle strength and muscle mass are associated with increased oxidative stress. Therefore, we measured serum protein carbonyl concentrations, muscle mass and strength, volume status and nutritional status and prospectively investigated whether these factors could be independent predictors for cardiovascular and all-cause mortality in patients undergoing HD.

## Methods

### Study subjects

This was a follow-up cross-sectional study on patients who were receiving thrice-weekly HD for at least 3 months at Hallym University Sacred Heart Hospital. We enrolled the patients undergoing HD with the same type of dialyzer and all patients were on high-flux dialysis using polysulfone membrane. Patients were excluded if they had a history of acute infection or hospitalization within 3 months before enrollment, had a medical condition that could affect serum protein carbonyl levels, such as liver cirrhosis, chronic inflammatory disease, or hematological or solid malignancies, or were taking immunosuppressants. The patients were recruited between April 2012 and August 2012.

The following baseline traditional cardiovascular risk factors were recorded at the start of the study: age, hypertension, diabetes mellitus, smoking history, and history of coronary artery disease (CAD), cerebrovascular disease (CVD) and peripheral artery disease (PAOD). Blood samples were collected to measure the following parameters immediately before a mid-week HD session: hemoglobin, blood urea nitrogen, creatinine, glucose, uric acid, albumin, 25-hydroxy vitamin D, total cholesterol, triglyceride, low-density lipoprotein (LDL) cholesterol, high-density lipoprotein (HDL), iron, transferrin, ferritin, fibrinogen, and intact parathyroid hormone concentrations. The cumulative doses of intravenous iron received during 6 months preceding the blood sampling were recorded. The residual renal function was assessed by β_2−_microglobulin and loss of residual renal function (defined as urine output less than 200 ml per day) [25, 26]. Serum levels of high-sensitivity C-reactive protein (hs-CRP) and interleukin (IL)-6 were measured using methods previously described [27]. Serum protein carbonyls were measured using a commercial ELISA kit (Zentech PC Test, Protein Carbonyl Enzyme Immuno-Assay Kit; Zenith Technologies, Dunedin, New Zealand). The assay has a minimum detectability of 20 *p*mol/mg protein, which is well below the range found in healthy human controls. The intra-assay and inter-assay coefficients of variation (CVs) for protein carbonyl measurements were 7.4 and 11.2%, respectively.

### Nutritional assessment

Nutritional status was assessed with the subjective global assessment (SGA) and anthropometric measurements. Body mass index (BMI) was calculated by the following equation: BMI = weight / height^2^ (kg/m^2^) and triceps skinfold thickness was measured using a Lange skinfold caliper (Beta Technology Inc., Cambridge, MD, USA). As previously mentioned, SGA was determined using the 7-point score system based on patient’s medical history and physical examination [27]. Based on the SGA score, the patients were categorized into 3 groups: SGA A (SGA score 6–7, well nourished), B (SGA score 3–5, mildly to moderately malnourished) or C (SGA score 1–2, severely malnourished). However, no patients were classified as SGA C in our cohort, and we compared SGA B patients with SGA A patients.

### Measurements of volume status, muscle mass and handgrip strength

Clinical assessment of dry weight was performed by our nephrologists. Clinical symptoms such as dyspnea, the absence or presence of peripheral edema, weakness and fatigue after HD, blood pressure and heart size on chest X-ray were used to estimate the patient’s dry weight. Measurements of body composition were performed using bioimpedance spectroscopy device (BIS, Body Composition Monitor; Fresenius Medical Care, Bad Homburg, Germany) as previously mentioned [28]. Low lean tissue index (LTI) was defined as an LTI below the tenth percentile of a reference population [29]. The total body water (TBW), intracellular water (ICW), extracellular water (ECW) and overhydration index (OH) were obtained, and overhydration status was defined as OH/ECW > 15% [30–32].

For the determination of muscle strength, handgrip strength (HGS) was measured using a handheld dynamometer (Jamar Plus+; Sammons Preston, Inc., Bolingbrook, IL, USA). HGS was measured three times on the non-fistula hand and each test was conducted after a 1-min rest, and the average value was obtained. Low muscle strength was defined as an HGS < 30 kg and < 20 kg in males and females, respectively. Sarcopenia was defined as low muscle mass and low muscle strength.

### Follow-up and long-term outcomes

Patient outcomes were observed until August 2018 and all patients were monitored regularly at our HD center. The long-term outcomes were all-cause and cardiovascular mortality. Cardiovascular mortality was defined as death with myocardial ischemia or myocardial infarction, congestive heart failure, significant arrhythmia, cardiac arrest and cerebrovascular accident.

### Statistical analysis

Statistical analyses were performed using SPSS ver. 25.0 (IBM Corp., Armonk, NY, USA). The Kolmogorov-Smirnov test was used to analyze the normality of distributions. Natural log values were used for skewed data, including protein carbonyl, IL-6, and hs-CRP levels. After log-transformation, we confirmed that these transformed values formed a normal distribution. Non-normally distributed variables are expressed as median scores and interquartile ranges (IQRs), whereas normally distributed variables are expressed as the mean scores with standard deviations. Protein carbonyl was examined both as a continuous variable and categorically in quartiles. Pearson’s correlation coefficient was used to evaluate possible relationships between log-protein carbonyl levels and possible variables. Multiple linear regression analysis was used to evaluate the association between plasma log-protein carbonyl levels and predictor variables. We compared patients with protein carbonyl levels in the highest quartile with those with levels in the 3 lowest quartiles, and increased oxidative stress was defined as displaying protein carbonyl levels in the highest quartile. Associations between increased oxidative stress and predictor valuables were analyzed by logistic regression analysis. The results are shown as odds ratios (ORs) with 95% confidence intervals (CIs). Survival curves were plotted using the Kaplan–Meier method and assessed by the log-rank test. The hazard ratios (HRs) for all-cause mortality were determined via Cox regression analyses and are presented with 95% CIs. Multivariate Cox proportional hazard models (forward stepwise selection) were used to evaluate adjusted HRs for the association between serum protein carbonyl and mortality. Because the present study included the relatively small number of patients, power analysis was performed. The model was adjusted for potential confounding factors including age, sex, diabetes, cardiovascular disease including CAD, CVD and PAOD, BMI and dialysis vintage. Statistical significance was set at *p* < 0.05.

## Results

### Patient characteristics

**Fig. 1:**
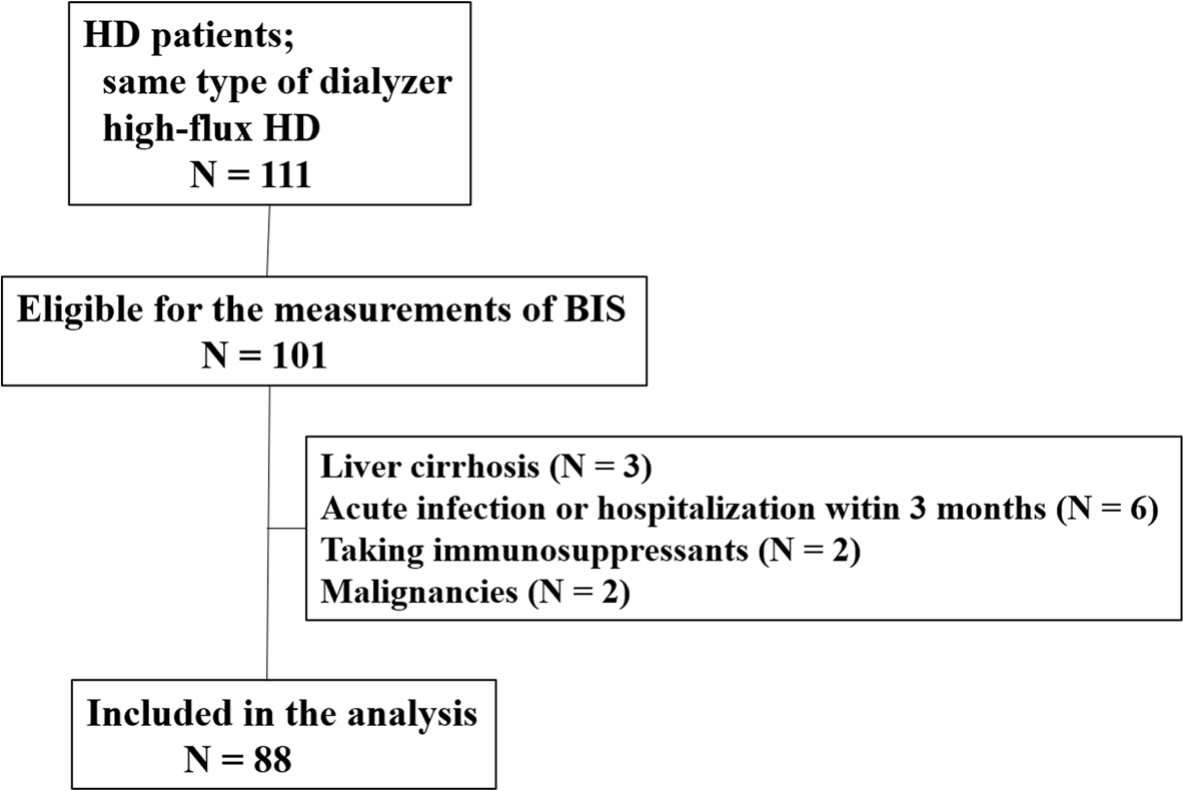
Patient population included in this analysis. BIS, bioimpedance spectroscopy; HD, hemodialysis

As shown in Fig. 1, eighty-eight patients undergoing HD were enrolled and followed prospectively for up to 6 years. Table 1 shows clinical data for patients. The patient mean age was 60.6 ± 13.5 years, and 50 (56.8%) were men. The mean duration of HD was 50.8 ± 41.3 months, and 50 of the patients (56.8%) had diabetes. The median serum protein carbonyl level was 79.0 (range 25.0–432.0) pmol/mg protein. During the mean follow-up period of 5.2 years, 30 (34.1%) patients died, and the median time to death was 29.5 (range 5.2–70.0) months. A total of 13 patients died from cardiovascular disease, 15 died from infection, 2 died from malignancy.

**Table 1:** Baseline characteristics of study patients

Variable	Total (***n*** = 88)
Age (years)	60.6 ± 13.5
Gender, male, n (%)	50 (56.8)
Diabetic, n (%)	50 (56.8)
Cause of ESRD, n (%)	
Diabetes	48 (54.5)
Hypertension	21 (23.9)
Glomerulonephritis	11 (12.5)
Others	8 (9.1)
MAP (mmHg)	103.9 ± 11.0
Dialysis vintage (months)	50.8 ± 41.3
HD access type, n (%)	
Arteriovenous fistula	53 (60.2)
Arteriovenous graft	35 (39.8)
CAD, n (%)	27 (23.8)
CVD, n (%)	21 (23.9)
PAOD, n (%)	7 (8.0)
Hemoglobin (g/dL)	9.7 ± 1.5
Glucose (mg/dL)	110.6 ± 63.3
Albumin (g/dL)	3.4 ± 0.4
Prealbumin (mg/dL)	26.6 ± 7.7
Uric acid (mg/DL)	7.8 ± 2.1
25-OH Vitamin D (ng/mL)	9.6 ± 4.7
intact PTH (pg/mL)	177.2 ± 244.1
Iron (μg/dL)	73.1 ± 31.1
Transferrin (μg/dL)	192.3 ± 44.1
Transferrin saturation (%)	44.5 ± 21.7
Ferritin (ng/mL)	201.0 ± 118.0
Total cholesterol (mg/dL)	140.8 ± 36.7
LDL (mg/dL)	79.6 ± 26.8
HDL (mg/dL)	44.2 ± 10.9
Triglyceride (mg/dL)	90.2 ± 48.7
β2-microglobulin (mg/L)	26.3 ± 7.7
Kt/V	1.5 ± 2.4
nPCR	1.1 ± 0.3
hs-CRP (mg/L)	1.0 (0.4–2.6)

*MAP* mean arterial pressure, *CAD* coronary artery disease, *CVD* cerebrovascular disease, *PAOD* peripheral artery disease, *PTH* parathyroid hormone, *LDL* low-density lipoprotein, *HDL* high-density lipoprotein, *nPCR* normalized protein catabolic rate, *hs-CRP* high-sensitivity C-reactive protein

### Factors associated with serum protein carbonyl levels

**Fig. 2:**
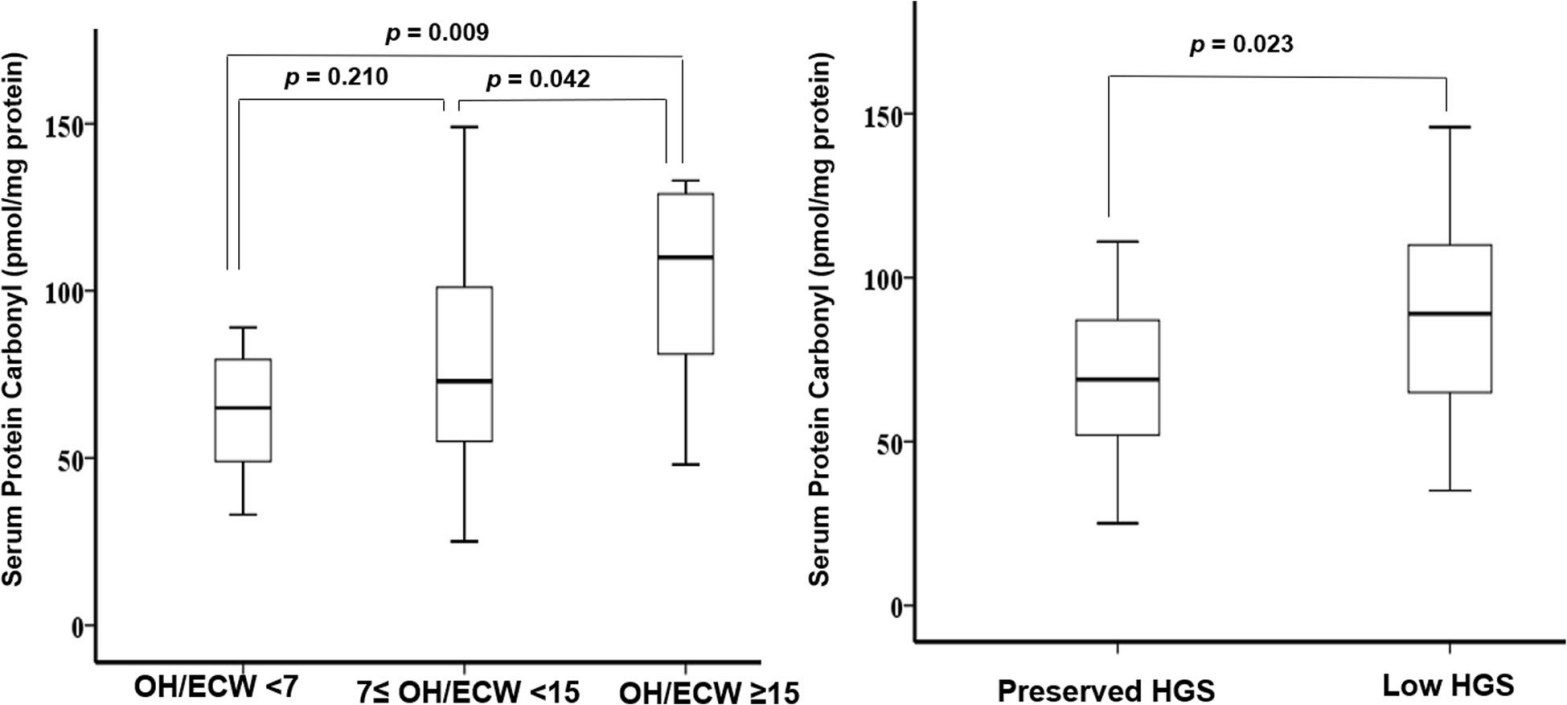
Box plots showing the difference in serum protein carbonyl levels according to volume status and muscle strength. The black lines in the box show the median values of the groups. The patients with overhydration and low HGS had significantly higher levels of serum protein carbonyl. OH/ECW, overhydration index/extracellular water; HGS, handgrip strength

Figure 2 shows that serum protein carbonyl levels were significantly higher in patients with overhydration and low HGS than in those with normohydration and preserved HGS, respectively.

The quartiles of serum protein carbonyl levels were as follows: quartile 1, 25.0 to < 57.5 pmol/mg protein; quartile 2, 57.5 to < 79.0 pmol/mg protein; quartile 3, 79.0 to 106.8 pmol/mg protein; quartile 4, 106.8 to 432.0 pmol/mg protein. Table 2 shows that patients with protein carbonyl levels in the highest quartile had significantly lower SGA scores (5.5 ± 0.8 vs. 6.1 ± 0.7, *p* = 0.001), lower serum level of prealbumin (22.1 ± 7.8 vs. 28.2 ± 8.6 mg/dL, *p* = 0.006) and higher OH/ECW values (11.5 ± 13.2 vs. -2.3 ± 13.6%, *p* < 0.001) than those with protein carbonyl levels in the 3 lower quartiles. HGS and LTI were significantly lower in men with protein carbonyl levels in the highest quartile than in men with levels in the 3 lower quartiles (23.6 ± 8.8 vs. 28.8 ± 7.6 kg, *p* = 0.042 and 12.0 ± 2.6 vs. 14.4 ± 3.2 kg/m^2^, *p* = 0.016, respectively) but not in females. BMI and FTI were not associated with serum protein carbonyl levels. The prevalence of overhydration (36.4% vs. 12.1%, *p* = 0.011) and low LTI (63.6% vs. 33.3%, *p* = 0.012) was significantly greater in patients with protein carbonyl levels in the highest quartile than in those with levels in the 3 lower quartiles.

**Table 2:** Clinical characteristics according to quartiles of protein carbonyl

Variable	Q1(***n*** = 22)	Q2 (***n*** = 22)	Q3(***n*** = 22)	Q4 (***n*** = 22)
	**25.0 to < 57.5**	**57.5 to < 79.0**	**79.0 to < 106.8**	**106.8 to 432.0**
Age (years)	56 (49–67)	59 (50–77)	63 (51–73)	60 (55–72)
Body mass index (kg/m^2^)	22 (20–24)	22 (21–24)	22 (19–25)	21 (20–24)
Gender, male, n (%)	12 (54.5)	11 (50.0)	12 (54.5)	16 (69.6)
Diabetic, n (%)	13 (59.1)	14 (63.6)	13 (59.1)	10 (43.5)
Cause of ESRD, n (%)				
Diabetes	13 (54.5)	13 (59.1)	13 (59.1)	10 (45.5)
Hypertension	6 (27.3)	4 (18.2)	6 (27.3)	5 (22.7)
Glomerulonephritis	2 (9.1)	2 (9.1)	3 (13.6)	4 (18.2)
Others	2 (9.1)	3 (13.6)	0	3 (13.6)
HD vintage (months)	40 (28–59)	19 (24–48)	20 (11–49)	38 (18–43)
CAD, n (%)	5 (22.7)	6 (27.3)	6 (27.3)	11 (50.0)
CVD, n (%)	2 (9.1)	6 (27.3)	6 (27.3)	7 (31.8)
PAOD, n (%)	1 (4.5)	2 (9.1)	1 (4.5)	3 (13.6)
Hemoglobin (g/dL)	9.7 (9.1–10.4)	10.6 (8.8–10.4)	10.1 (8.9–10.7)	9.7 (8.7–10.5)
Albumin (g/dL)	3.6 (3.5–3.7)	3.7 (3.4–3.7)	3.4 (3.0–3.6)	3.5 (3.3–3.6)
Prealbumin (mg/dL) *	30 (26–35)	25 (21–30)	26 (22–31)	23 (19–28)
Transferrin (μg/dL) *	217 (200–232)	200 (171–215)	185 (152–210)	175 (158–209)
Ferritin (ng/mL)	199 (86–309)	165 (125–237)	140 (92–251)	199 (139–312)
Transferrin saturation (%)	34 (28–40)	35 (28–51)	33 (24–42)	34 (30–42)
IV iron, n (%)	4 (18.2)	0	3 (13.6)	6 (27.3)
IV iron dose, mg	475 ± 75	0	400 ± 31	417 ± 31
Erythropoietin stimulating dose				
DPO (ug/week), *n* = 40	61 (35–98)	69 (48–92)	60 (39–93)	71 (48–101)
EPO (IU/week), *n* = 48	15,782	17,202	16,042	18,902
β2-microglobulin (mg/L)	24 (20–30)	26 (21–30)	17 (30–33)	29 (22–31)
HbA1c, (%)	6.8 (6.6–8.5)	6.4 (6.1–7.5)	6.8 (6.1–7.4)	7.2 (5.9–9.1)
Loss of RRF, n (%)	10 (45.4)	12 (54.5)	13 (59.1)	14 (63.6)
IL-6 (pg/ml)	3.1 (1.9–6.5)	5.3 (1.7–6.5)	5.7 (3.0–13.7)	5.4 (2.7–9.9)
hs-CRP (mg/L)	0.8 (0.6–1.9)	1.2 (0.3–6.5)	1.0 (0.6–2.1)	1.3 (0.5–3.8)
Fibrinogen (mg/dL)	326 (261–397)	339 (316–372)	3,329,267–374)	334 (261–417)
SGA score *	6.0 (6.0–7.0)	6.0 (6.0–7.0)	6.0 (5.0–7.0)	6.0 (5.0–6.0)
SGA < 6, n (%) *	2 (9.1)	4 (18.2)	6 (27.3)	10 (45.5)
OH/ECW (%) *	−1.5 (−11–29.)	−3.8 (−13.5–35)	0 (−16–58)	10.8 (3–48)
**HGS (kg)**				
Male *	30.5 (21–36)	31.6 (28–34)	29.3 (19–32)	23.6 (20–30)
Female	20.8 (20–26)	22.1 (18–27)	19.0 (16–22)	19.8 (19–20)
**LTI (kg/m**^**2)**^				
Male *	13.8 (11.8–16.2)	15.2 (11.5–16.9)	13.8 (11.5–16.2)	11.8 (9.3–14.3)
Female	11.3 (10.4–12.9)	12.6 (10.0–14.5)	11.3 (10.4–12.0)	11.1 (9.6–12.4)
FTI (kg/m^2)^				
Male	7.5 (5.5–14.5)	7.1 (3.0–10.8)	6.1 (4.6–10.5)	8.8 (7.1–10.1)
Female	9.3 (7.9–18.0)	7.6 (6.2–9.2)	11 (6.7–16.4)	12.0 (10.3–14.9)
Low LTI, n (%) *	8 (36.4)	7 (31.8)	7 (31.8)	14 (63.6)
Low HGS, n (%)	7 (31.8)	12 (54.5)	14 (63.6)	16 (72.7)
Mortality, n (%) *	5 (22.7)	6 (27.3)	6 (27.3)	13 (59.1)

*HD* hemodialysis, *CAD* coronary artery disease, *CVD* cerebrovascular disease, *PAOD* peripheral artery disease, *IV* intravenous, *DPO* Darbepoetin, *EPO* erythropoietin, *RRF* residual renal function, *hs-CRP* high-sensitivity C-reactive protein, *SGA* subjective global assessment, *OH* overhydration index, *ECW* extracellular water, *HGS* handgrip strength, *LTI* lean tissue index, *FTI* fat tissue index

*p < 0.05

As shown in Table 3, serum protein carbonyl levels were closely associated with serum levels of albumin (*r* = − 0.256, *p* = 0.016), prealbumin (*r* = − 0.309, *p* = 0.004) and transferrin (*r* = − 0.301, *p* = 0.004), SGA score (*r* = − 0.218, *p* = 0.014), OH/ECW (*r* = 0.295, *p* = 0.005) and low HGS (*r* = 0.243, *p =* 0.023).

**Table 3:** Correlation analysis of protein carbonyl level with clinical parameters

	Correlation coefficient	***p*** value
Albumin*	−0.256	0.016
Prealbumin*	−0.309	0.004
Transferrin*	−0.301	0.004
SGA score*	−0.218	0.014
OH/ECW*	0.295	0.005
Low HGS*	0.243	0.023
Low LTI	0.181	0.092
Transferrin saturation	−0.034	0.753
IV iron administration	0.193	0.071
Dialysis vintage	0.059	0.587
IL-6	0.184	0.092
Fibrinogen	0.024	0.823
hs-CRP	0.095	0.381
β2-microglobulin	0.162	0.136
Loss of RRF	0.034	0.755

*SGA* subjective global assessment, *OH* overhydration index, *ECW* extracellular water, *HGS* handgrip strength, *LTI* lean tissue index, *IV* intravenous, *RRF* residual renal function

*p < 0.05

A multivariate linear regression analysis demonstrated that the natural logarithm of the protein carbonyl level was associated with OH/ECW (Beta = 0.382, *p* = 0.001), albumin (Beta = − 0.291, *p* = 0.014), prealbumin (Beta = − 0.326, *p* = 0.008), transferrin (Beta = − 0.292, *p* = 0.011), overhydration (Beta = 0.393, *p* = 0.001), low HGS (Beta = 0.343, *p* = 0.006), low LTI (Beta = 0.227, *p* = 0.046) and sarcopenia (Beta = 0.237, *p* = 0.038) after adjusting for age, gender, diabetes, CAD, CVD, BMI and dialysis vintage. As shown in Table 4, significant predictors for increased oxidative stress were prealbumin (OR 0.91; 95% CI 0.83–0.99; *p* = 0.030), SGA category (OR, 3.52; 95% CI, 1.08–11.46; *p* = 0.037), overhydration (OR, 7.01; 95% CI, 1.77–27.79; *p* = 0.006), and sarcopenia (OR, 3.41; 95% CI, 1.02–11.32; *p* = 0.046).

**Table 4:** Factors for predicting increased oxidative stress (protein carbonyl ≥ Q4)

Variables	Univariate		Multivariate	
	OR (95% CI)	*P* value	OR (95% CI)	*P* value
Diabetes	0.54 (0.21–1.4)	0.217	0.45 (0.15–1.37)	0.157
CAD	2.62 (0.95–7.26)	0.064	2.45 (0.78–7.73)	0.127
CVD	1.86 (0.63–5.48)	0.262	1.53 (0.44–5.32)	0.506
Dialysis vintage	1.00 (0.99–1.02)	0.283	1.01 (0.99–1.02)	0.439
β2-microglobulin	1.02 (0.96–1.09)	0.485	1.01 (0.94–1.09)	0.740
Albumin	0.48 (0.14–1.70)	0.256	0.45 (0.10–2.02)	0.299
Prealbumin*	0.91 (0.85–0.98)	0.009	0.91 (0.83–0.99)	0.030
hs-CRP (log units)	1.32 (0.91–1.92)	0.150	1.51 (0.97–2.33)	0.066
Overhydration*	4.13 (1.32–12.96)	0.015	7.01 (1.77–27.79)	0.006
SGA (B vs. A)*	3.75 (1.32–10.68)	0.013	3.52 (1.08–11.46)	0.037
Low HGS	2.67 (0.93–7.66)	0.068	3.65 (0.99–13.51)	0.052
Low LTI*	3.50 (1.28–9.59)	0.015	4.63 (1.40–15.29)	0.012
Sarcopenia*	2.16 90.78–6.00)	0.138	3.41 (1.02–11.32)	0.046

Multivariate logistic analysis was performed after adjusting for age, gender, body mass index, diabetes, coronary artery disease, cerebrovascular disease, dialysis vintage

*CAD* coronary artery disease, *CVD* cerebrovascular disease, *hs-CRP* high-sensitivity C-reactive protein, *SGA* subjective global assessment, *HGS* handgrip strength, *LTI* lean tissue index

*p < 0.05

### Predictors for cardiovascular and all-cause mortality

**Fig. 3:**
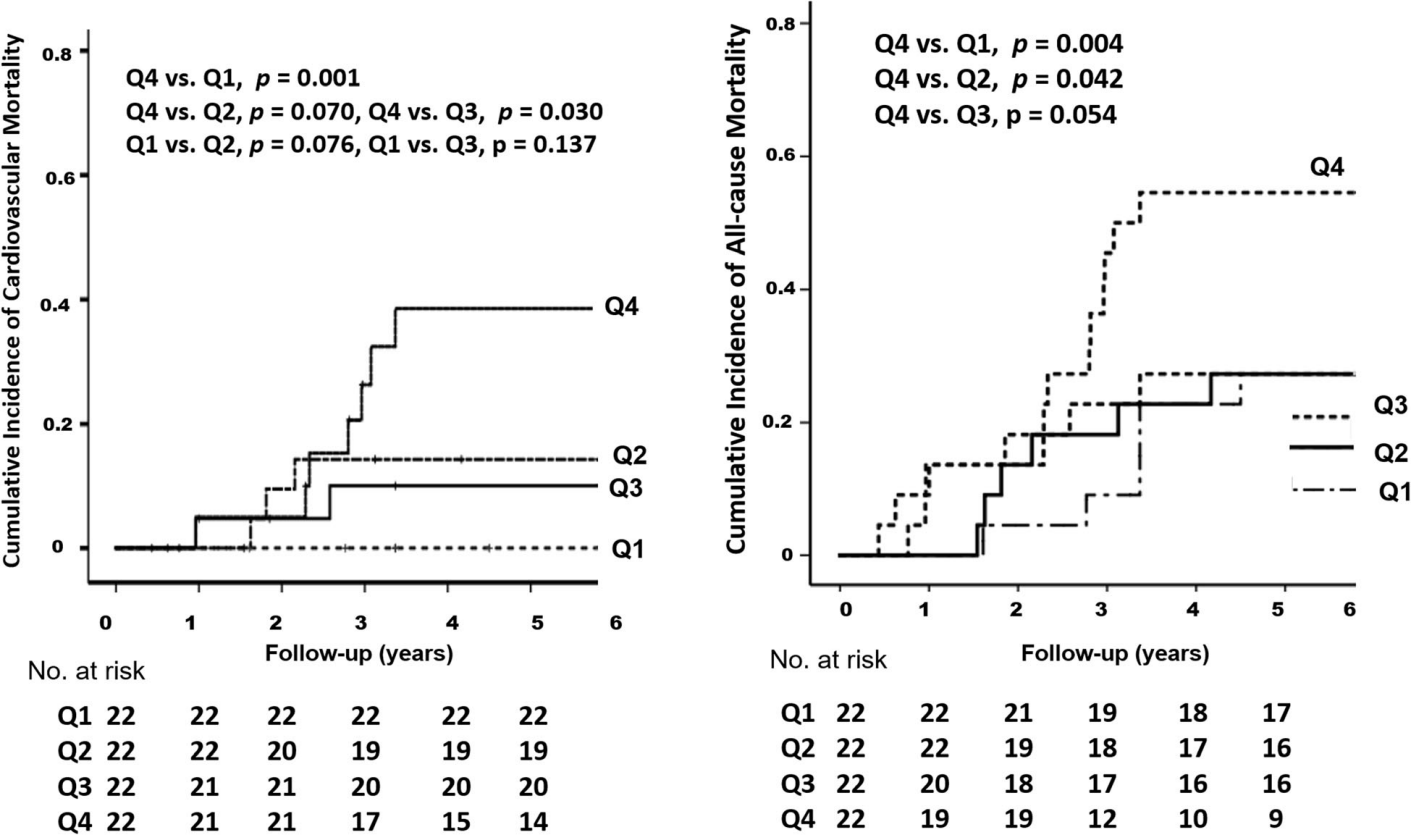
Kaplan-Meier estimates of survival according to the quartile of protein carbonyl levels. Patients with protein carbonyl levels in the highest quartile had the highest incidence of cardiovascular mortality and the poorest survival

Figure 3 graphically demonstrates the risks for cardiovascular and all-cause mortality according to baseline serum protein carbonyl quartiles. Multivariate analysis showed that patients with protein carbonyl levels in the highest quartile had a higher risk for all-cause mortality than those with levels in the lowest quartile (HR, 1.67; 95% CI, 1.05–2.68; *p* = 0.031), but the relationships of cardiovascular and all-cause mortality with protein carbonyl levels in the median quartiles were not significantly different from those in the lowest quartile. As shown in Table 5, age (HR, 1.07; 95% CI, 1.01–1.13; *p* = 0.014), CAD (HR, 4.45; 95% CI, 1.22–16.12; *p* = 0.024), serum levels of albumin (HR, 0.07; 95% CI, 0.16–0.37; *p* = 0.002) and prealbumin (HR, 0.85: 95% CI; 0.76–0.95; *p* = 0.003) and sarcopenia (HR, 7.71: 95% CI; 1.83–32.57; *p* = 0.018) were significant risk factors for cardiovascular mortality. In addition, each standard deviation increases in log-IL-6 (HR, 3.38; 95% CI, 1.16–7.22; *p* = 0.002) and log-protein carbonyl (HR, 6.90; 95% CI; 1.86–25.58; *p* = 0.004) levels significantly predicted cardiovascular mortality. In multivariate analysis for all-cause mortality, age (HR, 1.07; 95% CI, 1.03–1.11; *p* = 0.001), CAD (HR, 2.58; 95% CI, 1.10–6.72; *p* = 0.015), serum levels of prealbumin (HR, 0.86; 95% CI, 0.80–0.92; *p* = 0.001) and albumin (HR, 0.17; 95% CI, 0.06–0.46; *p* = 0.003), SGA category (HR, 6.51; 95% CI, 2.72–15.61; *p* < 0.001), overhydration (HR, 2.31; 95% CI, 1.26–8.71; *p* = 0.015) and sarcopenia (HR, 2.72; 95% CI, 1.11–6.63; *p* = 0.028) were significantly associated with mortality. In addition, each standard deviation increase in log-IL-6 levels (HR, 2.93; 95% CI, 1.83–4.69; *p* = 0.001), log-hs-CRP levels (HR, 1.89; 95% CI, 1.37–2.60; *p* < 0.001) and log-protein carbonyl levels (HR, 2.49; 95% CI, 1.08–5.75; *p* = 0.032) independently predicted all-cause mortality in HD patients.

**Table 5:** Multivariate analysis for cardiovascular and all-cause mortality in patients undergoing hemodialysis

	Cardiovascular		All–cause	
	HR (95% CI)	***P***	HR (95% CI)	***P***
**Age**	1.07 (1.01–1.13)	0.014	1.07 (1.03–1.11)	0.001
Gender (male vs. female)	1.12 (0.29–4.32)	0.870	0.67 (0.24–1.90)	0.453
Body mass index	1.11 (0.90–1.36)	0.348	1.01 (0.87–1.18)	0.904
Diabetes	1.05 (0.29–3.83)	0.723	1.52 (0.62–3.65)	0.363
**CAD**	4.45 (1.22–16.12)	0.024	2.58 (1.10–6.72)	0.015
CVD	1.42 (0.87–3.16)	0.420	0.64 (0.39–2.00)	0.318
PAOD	3.89 (1.01–18.41)	0.043	2.11 (0.94–20.8)	0.054
Dialysis vintage	1.01 (0.99–1.03)	0.256	1.00 (0.99–1.01)	0.613
**IL-6 (log units)**	3.38 (1.16–7.22)	0.002	2.93 (1.83–4.69)	0.001
**Hs-CRP (log units)**	1.75 (1.09–2.82)	0.021	1.89 (1.37–2.60)	< 0.001
**Albumin**	0.07 (0.16–0.37)	0.002	0.17 (0.06–0.46)	0.003
**Prealbumin**	0.85 (0.76–0.95)	0.003	0.86 (0.80–0.92)	0.001
**SGA (B vs. A)**	3.86 (1.87–23.87)	0.003	6.51 (2.72–15.61)	< 0.001
**Sarcopenia**	7.71 (1.83–32.57)	0.017	2.72 (1.11–6.63)	0.028
**Overhydration**	3.43 (0.77–15.23)	0.184	2.31 (1.26–8.71)	0.015
**Carbonyl (log units)**	6.90 (1.86–25.58)	0.004	2.37 (1.02–5.55)	0.036

Multivariate logistic analysis was performed after adjusting for age, gender, BMI, diabetes, CAD, CVD, PAOD and dialysis vintage

*CAD* coronary artery disease, *CVD* cerebrovascular disease, *PAOD* peripheral artery disease, *hs-CRP* high-sensitivity C-reactive protein, *SGA* subjective global assessment

## Discussion

In this study, we found that increased serum protein carbonyl levels were associated with overhydration, sarcopenia, SGA-assessed malnutrition and serum levels of prealbumin, and each standard deviation increase in log-protein carbonyl levels independently predicted all-cause and cardiovascular mortality after adjusting for age, sex, diabetes, CAD, CVD, BMI, dialysis vintage and serum albumin concentration in patients undergoing regular HD.

Patients with ESRD have accelerated cardiovascular morbidity and mortality rates due to extensive atherosclerosis, and this advanced atherosclerosis is caused by the increased production of reactive oxygen species in atheroma [6]. Increased oxidative stress is regarded as an important nontraditional uremia-specific risk factor [3]. Undergoing HD is associated with high levels of oxidative stress, and HD can further increase oxidative stress, explaining the high incidence of CVD in these patients [8]. In a cross-sectional cohort study, multiple biomarkers of inflammation and oxidative stress were higher in patients with stage 3 to 5 CKD, as well as in patients with ESRD, than in healthy subjects [3]. Another cross-sectional study showed that increased oxidative stress predicted severe coronary artery calcification scores in patients with ESRD [12]. However, studies examining the relationship between oxidative stress and hydration status and sarcopenia are limited in HD patients.

In the present study, we measured serum levels of protein carbonyl as a biomarker of protein oxidation. Protein carbonylation is one of irreversible oxidative protein modifications and is considered as an early marker of protein oxidative stress-related disorders. Protein carbonyls can be formed from metal-catalyzed oxidation of lysin, proline, arginine and threonine residues, direct oxidation of tryptophan and reactive lipid peroxidation products of cysteine, histidine and lysine. Because carbonylated proteins cannot be repaired by cellular enzymes, modified proteins must be degraded by the cell’s proteasome system [33]. The protein carbonyl level is accepted as a gold standard for measuring protein oxidation [20] and increases in parallel with CKD severity but decreases following renal transplantation and l-carnitine supplementation [3, 34, 35]. Uremia is associated with increased protein carbonyl stress and HD procedure can increase protein carbonyl level [36, 37]. To our knowledge, our study is the first to demonstrate the close relationship of the protein carbonyl level with overhydration and sarcopenia. In addition, we found that log-protein carbonyl levels were significantly correlated with serum levels of prealbumin, albumin and transferrin. This negative association between serum albumin and protein carbonyl levels supports the well-established theory that a low serum albumin level reflects the presence of systemic inflammation and oxidative stress as well as malnutrition or malabsorption [38]. Importantly, albumin is an important extracellular antioxidant molecule [22].

A close relationship between oxidative stress and sarcopenia has been demonstrated in the elderly population [39], but this relationship has not been well evaluated in HD patients. Sarcopenia is very prevalent and an important predictor of morbidity and mortality in CKD patients [40–42]. Although the pathophysiology of muscle aging is not completely understood, chronic inflammation, oxidative stress and mitochondrial dysfunction has been suggested to be associated with sarcopenia [43]. In the present study, we found that sarcopenia was associated with increased serum protein carbonyl levels in HD patients. Our findings suggest that oxidative stress is a strong cofactor for the development of adverse CV complications related to atherosclerosis, malnutrition, overhydration, sarcopenia and inflammation.

The achievement of normal volume status is an important goal of dialysis therapy, and overhydration is the most common cause of hypertension, significantly affecting poor cardiovascular outcomes and all-cause mortality in dialysis patients [30, 31, 44–46]. In accordance with previous reports, overhydration was a significant risk factor for mortality in our study. Whole-body bioimpedance spectroscopy is thought to be an objective method to assess volume status in dialysis patients and has now been widely used in clinical settings. Volume overload is well documented to be associated with malnutrition and chronic inflammation [47, 48], but its relationship with oxidative stress has been little investigated in dialysis patients. Oxidative stress has been suggested as a mechanism of chronic volume overload-induced cardiac dysfunction [49]. High sodium intake has been closely associated with oxidative stress and endothelial dysfunction in animal and human studies [50, 51], and lowering dialysate sodium improved endothelial dysfunction and oxidative stress in HD patients [52]. We found that volume overload was associated with increased oxidative stress in patients undergoing HD, suggesting that oxidative stress could be a pathogenesis of overhydration-associated complications.

The close relationship between diabetes and oxidative stress is well-documented [53]. In our study, pre-HD protein carbonyl levels are not significantly different in diabetic and nondiabetic HD patients. These results are consistent with the previous reports [36, 54]. Dursun and colleagues reported that both diabetes and HD increase oxidative stress, pre-HD protein carbonyl levels are not different in diabetic and non-diabetic patients [54]. Recent study measuring protein carbonyl levels before and after HD demonstrated that pre- and post-HD protein carbonyl levels were not different in nondiabetic and diabetic HD patients [36].

Our study has several limitations. First, there were relatively few patients in this study, but in the multivariate cox analysis for all-cause mortality, the estimated power of the study was 98.3%. Second, although protein carbonyl is a widely used biomarker of oxidative stress, this may not be sufficient to demonstrate oxidative stress, and we did not measure antioxidant levels. Third, we could not assess carbonyl stress induced by HD because we only measurd pre-HD protein carbonyl level.

## Conclusions

In conclusion, serum protein carbonyl levels are associated with volume status, malnutrition and sarcopenia and may predict all-cause and cardiovascular mortality in patients undergoing chronic HD, even after adjusting for age, sex, comorbid conditions, BMI and dialysis vintage. However, a large prospective population-based study is required to establish the potential role of oxidative stress in patients undergoing HD.

## Data Availability

The datasets used and/or analyzed during the current study are available from the corresponding author on reasonable request.

## Abbreviations

HD: Hemodialysis
CKD: Chronic kidney disease
ESRD: End stage renal Disease
SGA: Subjective global assessment
BMI: Body mass index
OH: Overhydration index
ECW: Extracellular water
HGS: Handgrip Strength
BIS: Bioimpedance spectroscopy
LTI: Lean tissue index
FTI: Fat tissue index
CVD: Cerebrovascular disease
CAD: Coronary artery disease
PAOD: Peripheral artery disease
ORs: Odds ratios
HRs: Hazard ratios
CI: Confidence interval
